# Draft Genome Sequence of *Bacillus subtilis* Strain NKYL29, an Antimicrobial-Peptide-Producing Strain from Soil

**DOI:** 10.1128/genomeA.01140-14

**Published:** 2014-11-06

**Authors:** Yanbin Jiang, Haijin Xu, Ying Li, Hongbin Liu, Lei Yu, Mingqiang Qiao, Gang Liu

**Affiliations:** a China Animal Disease Control Center, Beijing, China; b College of Life Sciences, Nankai University, Tianjin, China; c Institute of Subtropical Agriculture, Chinese Academy of Sciences, Scientific Observing and Experimental Station of Animal Nutrition and Feed Science in South-Central, Ministry of Agriculture, Hunan Provincial Engineering Research Center of Healthy Livestock, Key Laboratory of Agro-Ecological Processes in Subtropical Region, Changsha, Hunan, China

## Abstract

*Bacillus subtilis* strain NKYL29 is an antimicrobial-peptide-producing strain isolated from the soil of Ranzhuang Tunnel in Hebei Province, China. Here, we present the draft genome of this strain, which provides the genetic basis for application of the antimicrobial peptide.

## GENOME ANNOUNCEMENT

*Bacillus subtilis* has been used for agronomical purposes because it is safe, has a widespread distribution, promotes animal growth, produces compounds, and can survive in adverse habitats (1–4). *B. subtilis* is also considered a potential biological control agent due to its production of a variety of antimicrobial peptides that inhibit the growth of animal or plant pathogens. Most of the antimicrobial peptides produced by bacilli are ribosomal or nonribosomal peptides that exhibit resistance to high temperature, unfavorable pH, and proteolytic hydrolysis (5, 6).

The *B. subtilis* strain NKYL29 is isolated from the soil of Ranzhuang Tunnel in Hebei Province, China. The strain produces an unknown antimicrobial peptide and shows strong *in vitro* antimicrobial activity toward animal pathogens such as enterotoxic *Escherichia coli* and *Salmonella enteritidi*. To facilitate our research and application of this antimicrobial peptide, we performed genome sequencing of the NKYL29 strain.

Whole-genome shotgun sequencing of *B. subtilis* NKYL29 was performed at the Bioseeker Company, China, using the Illumina CASAVA pipeline version 1.8.3. SOAPdenovo version 2.04 was applied to assemble the raw FASTQ sequences into 96 contigs with a total length of 3.95 Mb (about 250× coverage) (7). The contigs were assembled into 22 scaffolds using the SSPACE Premium scaffolder version 2.2 (8). Annotation was done using the NCBI Prokaryotic Genome Annotation Pipeline and the Rapid Annotation Using Subsystem Technology (RAST) server (9, 10).

The genome sequence of *B. subtilis* NKYL29 comprises 3,942,386 bp, with an average GC content of 46.33%. The annotation revealed 4,009 open reading frames (ORFs), 4 rRNA genes, and 42 tRNA genes. The genome was then analyzed for the presence of genes encoding antimicrobial peptide synthetases, and 17 genes that show similarity to bacitracin stress-response genes were revealed. Among these genes, four genes show similarity to *bceA*, *bceB*, *bceR*, and *bceS*, respectively, and these genes form a *BceRS* two-component regulatory system that induces expression of the bacitracin transporter (11); there are also 3 genes similar to the LiaFSR two-component system of *B. subtilis*, which exhibits an immediate and graded response to the inducer bacitracin in the exponential growth phase (12). These specific ABC transporters and their associated genes may be induced in response to a nonribosomally synthesized antimicrobial peptide by *B. subtilis*, which inhibits cell-wall biosynthesis by binding very tightly to the long-chain undecaprenyl pyrophosphate (13, 14).

The availability of the *B. subtilis* NKYL29 draft genome sequence provides opportunities for biotechnological exploitation of genome features regarding the production of antimicrobial peptides.

### Nucleotide sequence accession numbers.

This whole-genome shotgun project has been deposited at DDBJ/EMBL/GenBank under the accession number JPYY00000000. The version described in this paper is version JPYY01000000. The strain is available from Haijin Xu (College of Life Sciences, Nankai University, Tianjin, China).
